# Optimized Design of the Basic Structure of Dry-Coupled Shear Wave Probe for Ultrasonic Testing of Rock and Concrete

**DOI:** 10.3390/s25092660

**Published:** 2025-04-23

**Authors:** Yonghao Lu, Yinqiu Zhou, Chenhui Zhu, Xueshen Cao, Hao Chen

**Affiliations:** 1 Institute of Acoustics, Chinese Academy of Sciences, Beijing 100190, China; luyonghao@mail.ioa.ac.cn (Y.L.); zhuchenhui@mail.ioa.ac.cn (C.Z.); caoxueshen@mail.ioa.ac.cn (X.C.); chh@mail.ioa.ac.cn (H.C.); 2 University of Chinese Academy of Sciences, Beijing 100049, China

**Keywords:** double-laminated, dry coupling, shear horizontal wave, dimension optimization

## Abstract

Although shear horizontal waves have advantages over longitudinal waves, including a higher resolution, less wave mode conversion, and much better reflection coefficients at void and crack interfaces in nondestructive detection, they require good contact surface flatness and efficient coupling agents. In this paper, we analyze and design the basic components of the dry-coupled ultrasonic shear wave probe through theoretical analyses and numerical simulations. The admittance characteristics, resonant frequency, and electromechanical coupling coefficients of the double-laminated vibrator under different size parameters in both 2D and 3D models are simulated, and the probe structures are optimized based on the simulation results and operational requirements. The simulation results of the wave field excited by the double-laminated vibrator show the effectiveness of the optimized probe models. Additionally, the dry coupling method of the probe is simulated to study the acoustic energy distribution under various dry-coupled structures. Finally, we compare the measured admittance with the simulated values, and they are in good agreement.

## 1. Introduction

Underground engineering encompasses the construction of mineral extraction tunnels, subterranean railway tunnels, and various other projects that are primarily characterized by their considerable magnitude and complex structure. The focus of subsurface engineering studies is on rock masses, where the stress–strain field has been changed due to the sustained influence of geological formations and external forces, causing the development and expansion of extensive cracks inside the rock. The configuration of these cracks significantly influences the macromechanical properties and failure characteristics of the rock [1]. Consequently, the detection of cracks in the rock and the research of their fracture evolutions hold substantial theoretical and practical significance for evaluating the safety and stability of subsurface engineering structures. In another research field, concrete is frequently utilized in the construction of structures such as residences, railways, highways, and water conservancy projects due to its exceptional plasticity, durability, and stability. The quality of concrete is directly linked to the safety of structures. However, under the influence of environmental factors and prolonged loads, concrete structures inevitably develop various defects, including cracks, voids, and spalling, thereby endangering the structural safety [2,3]. Therefore, the precise and effective detection of these internal defects is quite critical.

Common non-destructive testing methods [4] include ultrasonic pulse echo, impact echo [5], ground penetrating radar [6], and infrared thermography [7], which aim to identify structural defects to protect the internal stability. The ultrasonic pulse echo technique is widely used in exploration engineering for the detection of cracks in rocks and concretes due to its simple testing procedure, cost-effectiveness, and ease of result interpretation [8]. The shear wave has shorter wavelengths and a higher resolution than the longitudinal wave at the same frequency, making it more effective for larger-scale structure detections. The SH0 wave is entirely non-dispersive in the isotropic medium. The propagation of the SH0 wave is less affected by the surrounding media since its particle vibration direction is perpendicular to the wavefront [9]. Additionally, there is less mode conversion when the SH0 wave encounters defects or boundaries [10]. These characteristics of the SH0 wave can reduce the complexity of recorded signals and can facilitate the interpretation of results.

Traditional ultrasonic testing probes are primarily surface-coupled with large flat surfaces. However, materials such as rocks and concrete often have uneven and rough surfaces, so they require complicated processes like grinding and polishing the contact surfaces before testing. It is also necessary to apply appropriate coupling agents on the surface to fill air gaps, which can significantly minimize the coupling loss, as even minimal air gaps can lead to transmission losses of approximately 40 to 60 dB [11]. In some cases, surface cleanliness must be considered, and the prolonged use of numerous coupling agents is cumbersome and inconvenient. There are even particular circumstances where the use of coupling agents is prohibited. Especially for shear wave probes, high-viscosity coupling agents have the potential to make test signals unstable, easily resulting in signal loss [12]. To solve these problems, non-contact measurement methods are required. In the past, many experts have conducted research on different non-contact measurement methods, such as air-coupled ultrasound [13,14], laser ultrasonic [15,16], and electromagnetic ultrasonic [17,18]. However, the air-coupled probe has a rapidly attenuating signal and a narrow bandwidth; the laser ultrasonic equipment has low sensitivity; and the electromagnetic probe has low conversion efficiency [19]. In consequence, a dry coupling technique is required for materials like rock and concrete to enable non-contact detection while maintaining reliable ultrasonic transmission.

Since the 1960s, studies on the dry coupling technique of piezoelectric transducers have been conducted in the field of non-destructive testing [20]. Edwards tested the properties of different types of rubber and proposed a new dry couplant, which was expected to be used in the design of dry-coupled and air-coupled transducers [21]. Ravindran used a solid couplant based on silicone rubber to conduct dry-coupled ultrasonic pulse-echo testing on the nozzle throat of solid rocket boosters at the optimal detection frequency of 1 MHz, which effectively avoided contamination of the graphite material by liquid couplant [22]. Robinson developed a low-frequency dry coupled wheel probe to inspect adhesively bonded joints, using a high-damping property F28 rubber as the dry-coupling material [23]. Augereau designed a probe that uses a silicon rod as a dry coupling material, which was capable of achieving high-precision measurement of elastic parameters of the 6061-T6 aluminum alloy at temperatures up to 220 degrees [24]. In the field of concrete detection, Shevaldykin investigated a dry point contact ultrasonic transducer for concrete defect detection; its acoustic contact size from the front end of the probe to the contact surface is 1–2 mm, which is much smaller than the typical value of the concrete wave field. Thus, it can be approximately regarded as the point contact on the contact surface. In this case, coupling agents have nearly no influence on the coefficient of signal transformation and become useless [25].

The earliest inspections of large concrete structures required the continuous application of a significant quantity of coupling agent until the inspection was completed, which is both inconvenient and costly. Moreover, concrete structures often have irregular surfaces, causing inadequate contact between the probe and the detecting surface, which leads to considerable losses of acoustic energy. Similarly, this problem also occurs in rock detections. Based on previously reported research, we propose a dry-coupled shear wave probe structure with point contact, which can address these two problems in the detection. The basic composition of the probe includes the dry coupling head, the piezoelectric vibrator, and the damping layer. The dry coupling head achieves point contact through the hemispherical shape, and it meets the acoustic impedance matching as much as possible between the piezoelectric vibrator and the test object. Additionally, the vibrator consists of two identical piezoelectric ceramic plates, and the flexural vibration of the double-laminated vibrator can excite low-frequency shear waves. This paper focuses on the optimized design of the two-dimensional and three-dimensional models of the double-laminated vibrator under the flexural vibration mode. By comparing the differences between theoretical and simulated values, along with the relationships of resonant frequency and electromechanical coupling coefficient with variations in length, thickness, width, and structural shape, we select suitable structures and dimensions for the detection frequency of rocks and concretes. Additionally, we conduct a brief simulation and analysis of the two-dimensional and three-dimensional wave fields generated by the vibrator in the time domain, revealing propagation characteristics that are conducive to shear wave detection. Furthermore, the discussion on acoustic energy with different coupling methods is based on simulation results, that the energy of the dry point coupling is relatively weak but can be increased by parallel excitation through an array. Ultimately, we bond the customized piezoelectric plates into a double-laminated vibrator and measure its admittance curve. The results are in good agreement with the simulation values, preliminarily proving the reliability of the simulation work to some extent.

## 2. Materials and Methods

### 2.1. Flexural Vibration of a Long Strip Rectangular Double-Laminated Piezoelectric Ceramic Vibrator

**Figure 1:**
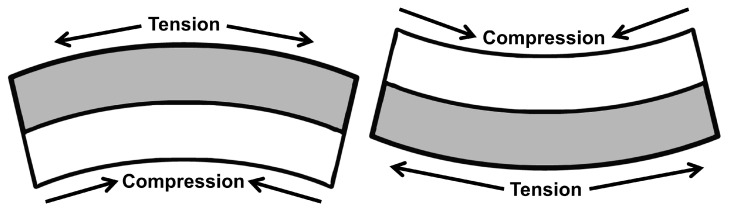
State of flexural vibration of the rectangular piezoelectric double-laminated vibrator.

Flexural vibration piezoelectric ceramic vibrators have very numerous applications in piezoelectric ceramic filters, air ultrasonic transducers, and underwater ultrasonic transducers because the flexural vibration piezoelectric ceramic vibrator makes it easy to achieve acoustic matching with media like air and water due to its low radiation impedance. However, it also produces much lower resonant frequencies than longitudinal and thickness vibration frequencies under the same geometry. Figure 1 illustrates the operation principle of the piezoelectric ceramic double-laminated vibrator’s flexural vibration. At a certain moment under the excitation of an electric field, when one of the piezoelectric elements extends, the other one contracts, which results in the all of the rectangular elements producing flexural vibration modes.

The fundamental equation for the flexural vibration of a piezoelectric double-laminated rod with a rectangular cross-section is shown as follows [26]:

(1) $$\frac{\partial^{2}u}{\partial t^{2}}-r^{2}\frac{\partial^{4}u}{\partial x^{2}\partial t^{2}}+\frac{Er^{2}}{\rho }\cdot \frac{\partial^{4}u}{\partial x^{4}}=0,$$

where u is vibration displacement, r is the radius of gyration, E is Young’s modulus, and ρ is the material density. When the cross-sectional dimension of the piezoelectric vibrator is much smaller than its length, the wavelength of the flexural vibration is much larger than the cross-sectional dimension; thus, the second term in Equation (1) can be ignored compared to the first term, and the wave equation becomes the following:

(2) $$\frac{\partial^{2}u}{\partial t^{2}}+\frac{Er^{2}}{\rho }\cdot \frac{\partial^{4}u}{\partial x^{4}}=0.$$

Assuming the form of the solution of Equation (2) is u=cos(ωt+φ), and then the following is true:

(3) $$\frac{\partial^{4}u}{\partial x^{4}}=m^{4}u,$$

where $m^{4}=\omega^{2}\rho /Er^{2}$, and the general solution of Equation (3) is written as follows:

(4) u=(Acoshmx+Bsinhmx+Ccosmx+Dsinmx)cos(ωt+φ).

After introducing the constitutive relation of the piezoelectric equation, it can be obtained that when the mechanical boundary conditions are traction-free at both ends, the resonant frequency of the flexural vibration of the double-laminated rod is given by the following [26]:

(5) $$f_{r}=\beta_{r}^{2}\frac{\pi h}{4l^{2}\sqrt{3\rho s_{11}^{E}}},$$

where h is the whole thickness of the vibrator; l is the length of the vibrator; $s_{11}^{E}$ is the elastic compliance constants of the piezoelectric material; $\beta_{n}=(n+1/2)$, and n represents the order of the flexural vibration mode. For the flexural vibration of a rectangular piezoelectric double-laminated rod with both traction-free ends, the positions of the nodes where the displacement is zero at the fundamental frequency are symmetrical. The electrode leads should be positioned as close as possible to these two nodes, which ensures the reduction of vibration energy dissipation and improvements in the stability of the vibrator.

### 2.2. Flexural Vibration of a Finite-Width Rectangular Double-Laminated Piezoelectric Ceramic Vibrator

Previously, we discussed the analytical expression of the long strip rectangular double-laminated vibrator in a two-dimensional model. Next, we consider a three-dimensional model of a plate-like rectangular double-laminated vibrator with a finite width. By introducing the mechanical coupling coefficient [27], the total vibration of the three-dimensional model is approximated as two equivalent flexural vibrations of the long strip rectangular double-laminated vibrator, which are mechanically coupled together. Using this equivalent model, the resonant frequency equations for these two equivalent vibrations can be obtained, and finally, the approximate equation for the whole vibrator can be calculated.

**Figure 2:**
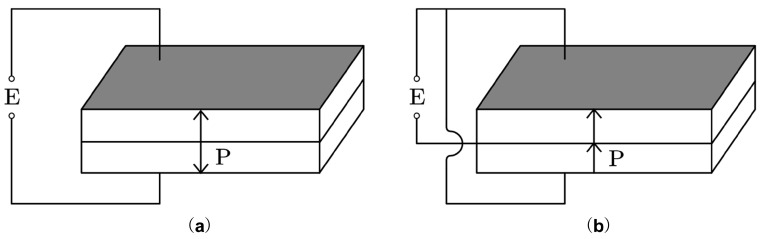
Schematic diagram of rectangular piezoelectric double-laminated vibrator: (**a**) connected in series, (**b**) connected in parallel.

Figure 2 shows that the flexural vibration mode can be excited when two rectangular piezoelectric ceramic plates with the same geometric dimensions and electrodes are bonded together. Polarization directions are either opposite while the vibrator is connected in series (Figure 2a) or the same while the vibrator is connected in parallel (Figure 2b).

**Figure 3:**
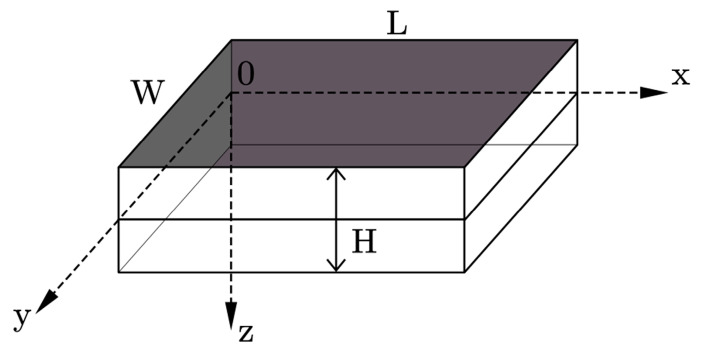
Geometric dimensions of rectangular piezoelectric double-laminated vibrators.

Figure 3 depicts the Cartesian coordinates and the geometrical dimensions of the rectangular piezoelectric double-laminated plate in flexural vibration; L, W, and H respectively represent the length, width, and thickness of the vibrator. According to the elastic dynamic theory, for the linear flexural vibration of a rectangular thin plate, the strain components can be expressed as the following Equations:

(6) $$\left\{\begin{array}{l} \varepsilon_{x}=-\left(\frac{\partial^{2}u}{\partial x^{2}}\right)z,\\ \varepsilon_{y}=-\left(\frac{\partial^{2}u}{\partial y^{2}}\right)z,\\ \varepsilon_{z}=-2\left(\frac{\partial^{2}u}{\partial x\partial y}\right)z, \end{array}\right.$$

In Equation (6), $u=u\left(x,y,t\right)$ is the flexural vibrational displacement of the vibrator. The piezoelectric equations describe the constitutive connection between stress and strain in piezoelectric ceramics, and the piezoelectric equation of a ceramic thin plate can be simplified as follows:

(7) $$\left\{\begin{array}{l} \varepsilon_{x}=s_{11}^{E}\sigma_{x}+s_{12}^{E}\sigma_{y}+d_{31}E_{z},\\ \varepsilon_{y}=s_{12}^{E}\sigma_{x}+s_{11}^{E}\sigma_{y}+d_{31}E_{z},\\ D_{z}=d_{31}\left(\sigma_{x}+\sigma_{y}\right)+\varepsilon_{33}^{T}E_{z}, \end{array}\right.$$

where $E_{z}$ and $D_{z}$ are the electric field and the electric displacement in the thickness direction of the vibrator, $s_{11}^{E}$ and $s_{12}^{E}$ are the elastic compliance constants, $\sigma_{x}$, $\sigma_{y}$ and $\varepsilon_{x}$, $\varepsilon_{y}$ are the stresses and strains in the length and width direction of the vibrator, $d_{31}$ is the piezoelectric strain constant, and $\varepsilon_{33}^{T}$ is the free dielectric constant component of the piezoelectric material. Let $n=\sigma_{x}/\sigma_{y}$, which is defined as the mechanical coupling coefficient. Using the expression of the mechanical coupling coefficient, Equation (7) can be rewritten as follows:

(8) $$\left\{\begin{array}{l} \varepsilon_{x}=\left(s_{11}^{E}+\frac{s_{12}^{E}}{n}\right)\sigma_{x}+d_{31}E_{z},\\ \varepsilon_{y}=\left(s_{11}^{E}+s_{12}^{E}n\right)\sigma_{y}+d_{31}E_{z}, \end{array}\right.$$

When the mechanical coupling coefficient is introduced, the flexural vibration of a thin plate can be reduced to two equivalent one-dimensional flexural vibrations of the rectangular double-laminated strip around the *x*-axis and the *y*-axis. In this case, the flexural vibrational displacement can be expressed as the following form:

(9) $$u=u\left(x,y,t\right)=u_{x}\left(x\right)u_{y}\left(y\right)\exp\left(j\omega t\right),$$

These two equivalent vibrations appear to be independent, but they are associated because of the Poisson effect. Consider the equations of flexural vibration around the *y*-axis (10) and *x*-axis (11) of a rectangular thin plate:

(10) $$-\frac{\partial^{2}u}{\partial x^{4}}=\frac{1}{c_{x}^{2}R^{2}}\cdot \frac{\partial^{2}u}{\partial t^{2}},$$

(11) $$-\frac{\partial^{2}u}{\partial y^{4}}=\frac{1}{c_{y}^{2}R^{2}}\cdot \frac{\partial^{2}u}{\partial t^{2}},$$

where $c_{x}^{2}={\left[\rho s_{11}^{E}\left(1-\upsilon /n\right)\right]}^{-1}$, $c_{y}^{2}={\left[\rho s_{11}^{E}\left(1-\upsilon n\right)\right]}^{-1}$, $\upsilon =-s_{12}^{E}/s_{11}^{E}$, ρ is the volume density of the material, and $R=H/\sqrt{12}$ is the cross-sectional gyration radius [27]. Equations (10) and (11) have general solutions and are shown as follows:

(12) $$u_{x}\left(x\right)=A_{x}\cosh\frac{\omega }{k_{x}}x+B_{x}\sinh\frac{\omega }{k_{x}}x+C_{x}\cos\frac{\omega }{k_{x}}x+D_{x}\sin\frac{\omega }{k_{x}}x,$$

(13) $$u_{y}\left(y\right)=A_{y}\cosh\frac{\omega }{k_{y}}y+B_{y}\sinh\frac{\omega }{k_{y}}y+C_{y}\cos\frac{\omega }{k_{y}}y+D_{y}\sin\frac{\omega }{k_{y}}y,$$

where $k_{x}=\sqrt{\omega c_{x}R}$, and $k_{y}=\sqrt{\omega c_{y}R}$. When the boundary conditions are free, the flexural moment and the shearing force at the boundary are zero, and the resonant frequency equations for the two equivalent one-dimensional flexural vibrations can be obtained as follows:

(14) $$\cosh\frac{\omega }{k_{x}}L\cdot \cos\frac{\omega }{k_{x}}L=1,$$

(15) $$\cosh\frac{\omega }{k_{y}}W\cdot \cos\frac{\omega }{k_{y}}W=1,$$

The solutions of these two equations are $R\left(i\right)$ and $R\left(j\right)$, and different combinations of positive integers i and j represent various orders of the flexural vibrations.

(16) $$\frac{\omega }{k_{x}}L=R\left(i\right),\;\;\;\;i=0,1,2,\cdots ,$$

(17) $$\frac{\omega }{k_{y}}W=R\left(j\right),\;\;\;\;j=0,1,2,\cdots ,$$

From these two solutions, we can obtain the equation of the mechanical coupling coefficient and the final expression of the resonant frequencies in Equations (18) and (19). It can be seen that, once the material, geometric dimensions, and flexural vibration orders of the rectangular piezoelectric ceramic vibrator are given, the resonant frequencies of two equivalent vibrations and the coupled vibration can be obtained as follows:

(18) $$\upsilon \frac{R^{4}\left(i\right)}{R^{4}\left(j\right)}n^{2}+\left[{\left(\frac{L}{W}\right)}^{4}-\frac{R^{4}\left(i\right)}{R^{4}\left(j\right)}\right]n-\upsilon {\left(\frac{L}{W}\right)}^{4}=0,$$

(19) $$f_{ij}=\frac{c_{r}R}{2\pi \sqrt{1-\upsilon^{2}}}\left[\frac{R^{2}\left(i\right)}{L^{2}}+\frac{R^{2}\left(j\right)}{W^{2}}\right],$$

### 2.3. Numerical Calculation Based on the Finite Element Method

The finite element method, which is one of the most effective approaches for the design of piezoelectric transducers, has become a widely adopted numerical calculation method in engineering. The finite element method does not require large-scale simplifications for transducers of any structure or in any operating state, allowing it to simulate the actual working conditions of transducers to the greatest extent. Its advantages include its capacity to handle complicated structures and boundaries, different external coupling methods, materials with various parameter properties, structures with and without mechanical losses, and other complex models.

The finite element method is based on the variational principle. The basic idea is to partition the solution domain into a finite number of interconnected element assemblies in a certain manner. Parameters at the nodes within the elements or at the boundaries of the elements are handled as unknowns, and the interpolation functions are constructed to relate the unknown node values to the values at any point within the elements, thereby establishing a system of equations that approximately satisfies the entire continuum.

## 3. Finite Element Simulation Results

### 3.1. Finite Element Analysis of Flexural Vibration of Rectangular Double-Laminated Vibrator

**Figure 4:**
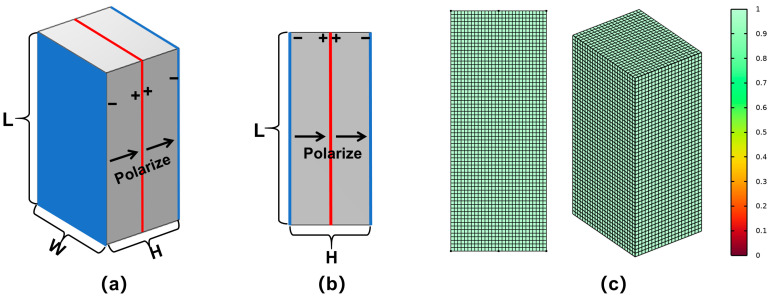
Rectangular double-laminated vibrator model: (**a**) 3D model of vibrator, (**b**) 2D model of vibrator, and (**c**) mesh quality of two models.

Firstly, the physical model of a rectangular piezoelectric double-laminated vibrator is established, and the material parameters of the piezoelectric are defined. Comsol 6.2 finite element simulation software was used to establish the 3D physical models, as shown in Figure 4a. L, W, and H respectively represent the length, width, and thickness of the double-laminated vibrator. The width of the piezoelectric plate is limited, but there is no transverse stress in the flexural vibration process, so the corresponding finite element calculation model can be simplified into a two-dimensional model, as shown in Figure 4b, thus saving calculation time. The mesh qualities of two-dimensional and three-dimensional models are shown in Figure 4c. Meanwhile, the calculation results of the two-dimensional model are compared to those of the three-dimensional model.

The piezoelectric vibrator material is PZT-4, and the polarization direction of two piezoelectric plates is the same along the thickness H direction. The surface of both sides of the piezoelectric vibrator is grounded while the common surface is set as a positive electrode. The circuit leads are connected with the external electric field in a parallel circuit connection. Under the excitation of the electric field, one of the vibrators is extended while the other one is shortened, thus producing flexural vibration, as shown in Figure 1. The parameters in Table 1 describing piezoelectric ceramic materials include elasticity matrix, relative permittivity, and coupling matrix.

#### 3.1.1. Two-Dimensional Model

**Figure 5:**
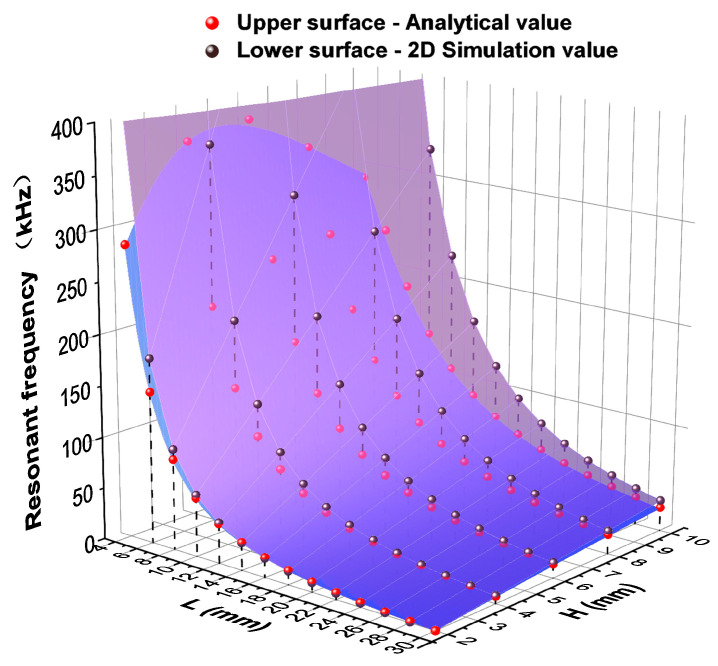
The comparison of the resonant frequency between two-dimensional model simulation solutions and analytical solutions.

Firstly, we studied the two-dimensional model in Figure 4b and used Comsol 6.2 to study the variation of the resonance frequency of the first-order bending vibration under different lengths and thicknesses, where 4 mm ≤ L ≤ 30 mm, 2 mm ≤ H ≤ 10 mm. We compared the simulation results with the numerical values of the analytical solution of the resonant frequency in Equation (5), as shown in Figure 5, where the upper surface represents the analytical values while the lower surface represents the two-dimensional simulation values. We intuitively observed that, in regions where the length is longer and the thickness is thinner, the differences between analytical and simulation values are smaller. Conversely, as the length decreases and the thickness increases, the differences become larger. Especially when the thickness of the double-laminated vibrator increases to 10 mm, the differences across the entire H = 10 mm section are at their maximum.

**Figure 6:**
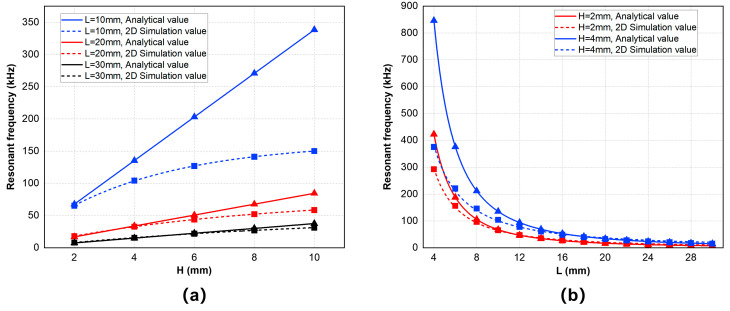
Analytical value and two-dimensional simulation value of resonant frequency with a certain length or thickness: (**a**) resonant frequency curve with H and (**b**) resonant frequency curve with L.

To compare the results more clearly, we listed some specific data of analytical values and two-dimensional simulation values under different lengths and thicknesses and calculated their relative errors in Table 2 and Table 3. Then we chose data points on the cross-sections of L = 10 mm, L = 20 mm, L = 30 mm, and H = 2 mm, H = 4 mm, H = 8 mm for the next analysis in Figure 6. Figure 6a shows the comparison between the analytical and simulated values of the resonant frequencies at different thicknesses with a certain length while Figure 6b shows the comparison at different lengths with a certain thickness. From these tables and figures, it can be concluded that, when the thickness of the whole double-laminated vibrator is thinner and the length is longer, the resonant frequency of its first-order flexural vibration mode corresponds more closely to the theoretical value given in Equation (5). However, when the value of H/L increases, the dimensions of the geometric model no longer satisfy the condition that the cross-sectional dimensions are much smaller than the length. At this time, Equation (1) for the flexural vibration of the rod-shaped rectangular cross-section double-laminated vibrator fails to be simplified; thus, Equation (5) is also no longer applicable for calculating the resonant frequency of flexural vibrations. However, both theoretical and simulation results show that the general rule is that the first-order flexural vibration resonant frequency of the piezoelectric double-laminated vibrator increases with thickness and decreases with length and changes more rapidly with length. The simulation results of the flexural vibration resonant frequency at different sizes effectively guide the design of the probe, indicating how to select the size of the double-laminated vibrator under different working frequency needs. For example, in concrete defect detection, the working frequency is often set anywhere from 50 to 100 kHz.

Meanwhile, the electromechanical coupling coefficient is calculated by the resonant and anti-resonant frequencies through the following Equation (20), where $f_{r}$ represents the resonant frequency, $f_{a}$ indicates the anti-resonant frequency, and both frequencies can be obtained by the simulation of the impedance curve:

(20) $$k_{e}=\sqrt{\left(f_{a}^{2}-f_{r}^{2}\right)/f_{a}^{2}},$$

**Figure 7:**
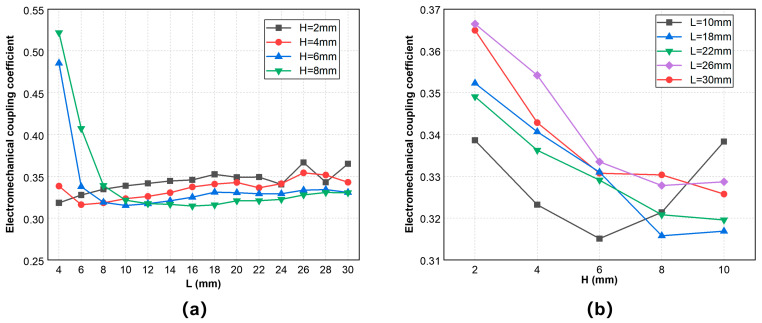
Electromechanical coupling coefficient curve of the vibrator with a certain length or thickness: (**a**) electromechanical coupling coefficient curve with L and (**b**) electromechanical coupling coefficient curve with H.

Figure 7 shows the simulation result of the electromechanical coupling coefficient of the vibrator at several geometric sizes. Figure 7a demonstrates that the electromechanical coupling coefficient generally shows an increasing trend with an increase of the vibrator length and a decrease of the vibrator thickness. As the value of H/L decreases, the geometric model of the vibrator is closer to a thin plate, so the electromechanical coupling coefficient increases. Conversely, when the vibrator plate is not thin enough, as shown on the left side of Figure 7a, the electromechanical coupling coefficient increases sharply. This is because the vibration mode of the double-laminated vibrator is no longer a pure flexural vibration but may be coupled with vibrations of other modes. Figure 7b also demonstrates the same trend of the electromechanical coupling coefficient with changes of the geometric sizes when the thickness H does not exceed 6 mm. However, models with longer lengths do not necessarily have larger mechanical coupling coefficients; for instance, the value of L = 26 mm is higher than the value of L = 30 mm, and the value of L = 18 mm is also higher than the value of L = 22 mm, which are also reflected in Figure 7a.

#### 3.1.2. Three-Dimensional Model

**Figure 8:**
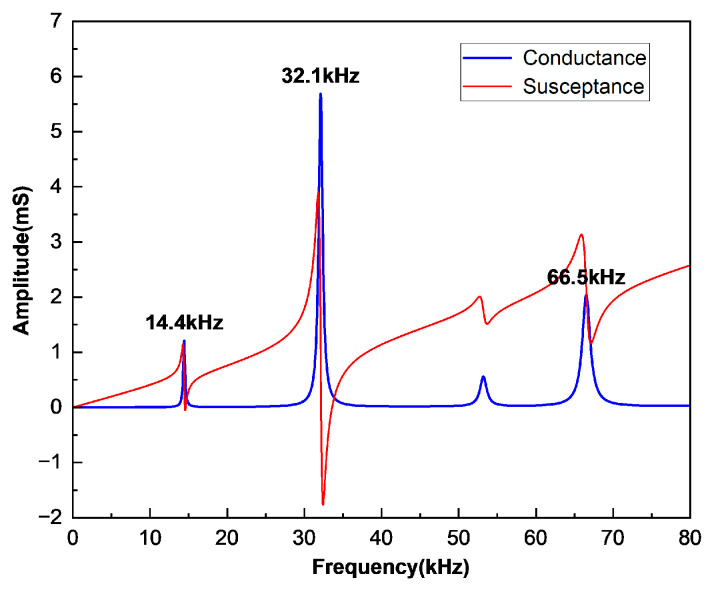
Conductance and susceptance curves of three kinds of first-order flexural vibration of the double-laminated vibrator.

**Figure 9:**
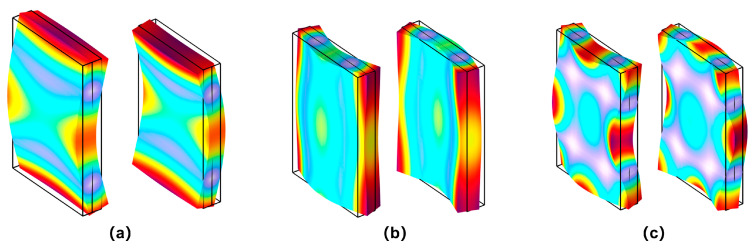
Three modes of first-order flexural vibration of the double-laminated vibrator: (**a**) flexural vibration around length, (**b**) flexural vibration around width, and (**c**) coupled flexural vibration around length and width.

The change in the three-dimensional model is the addition of the width dimension parameter. In theory, the parameter of the width has no effect on the two-dimensional model, which can be verified later by comparison. After considering the width dimension, the three-dimensional model of the vibrator has an additional mode of flexural vibration around the width direction. Furthermore, as mentioned in the theoretical part, the double-laminated vibrator can generate coupled flexural vibrations simultaneously around both length and width directions. The model parameters were set as L = 30 mm, W = 20 mm, and H = 4 mm to conduct the simulation, and the admittance curve within 80 kHz is shown in Figure 8. The resonance peak at 14.4 kHz corresponds to the first-order flexural vibration mode around length direction; the resonance peak at 32.1 kHz corresponds to the first-order flexural vibration mode around width direction; and the resonance peak at 66.5 kHz corresponds to their coupled vibration mode. Figure 9 shows three corresponding modes of vibration.

**Figure 10:**
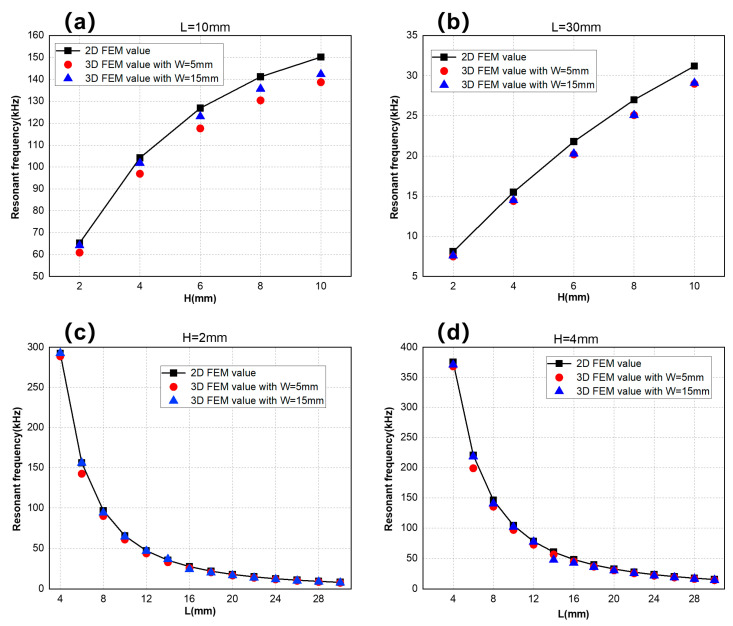
Simulation values of resonant frequencies of 2D and 3D models at a certain length, width, and thickness: (**a**) L = 10 mm, resonant frequency curve with H, (**b**) L = 30 mm, resonant frequency curve with H, (**c**) H = 2 mm, resonant frequency curve with L, and (**d**) H = 4 mm, resonant frequency curve with L.

Since two flexural vibration modes around length and width are similar, we only talk about the case of the first-order flexural vibration around length. We set two groups of model parameters with W = 5 mm and W = 15 mm, and, similarly to the analysis of the two-dimensional model, we selected the simulation values of H = 2 mm, H = 4 mm and L = 10 mm, L = 30 mm, as well as plotted the curves of resonant frequencies in Figure 10, comparing them with their simulation values of a two-dimensional model. Figure 10a,b shows the relationship between the resonant frequency and thickness of flexural vibrations around the length direction under two sets of length parameters and compares the simulation values with different widths. In Figure 10b, as the length increases, the impact of the width parameter on the resonant frequency of the three-dimensional model reduces; however, there are still differences between the simulation values in the three-dimensional model and those in the two-dimensional model, and these differences increase with either increasing thickness or decreasing width. The reason is that models with large thicknesses and small widths cannot be simplified into a plane problem, so the two-dimensional model is not applicable anymore. Figure 10c,d shows the same trend as before, but the differences are larger when W = 5 mm, L = 6 mm, and W = 15 mm, L = 14 mm. This is because when the model’s length and width are approximately equal, it is easier to generate coupled flexural vibrations, affecting the independent flexural vibration around the length direction.

**Figure 11:**
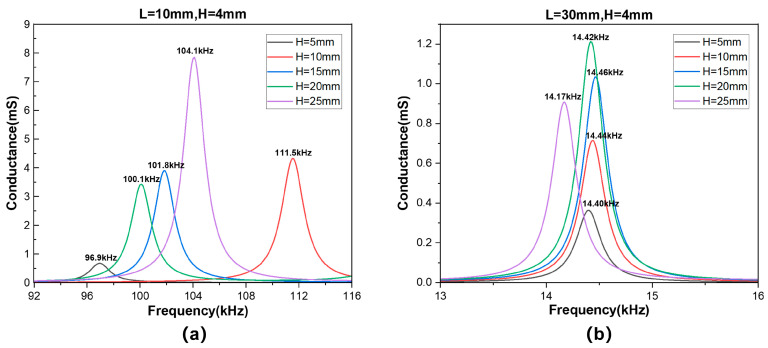
The curve of conductance with frequency at different widths: (**a**) L = 10 mm, H = 4 mm and (**b**) L = 30 mm, H = 4 mm.

Two groups of certain length and thickness values were selected to evaluate the variations in the resonant frequency of flexural vibrations around the length direction at different widths, as illustrated in Figure 11. Figure 11a demonstrates that, when the H/L value is insufficiently small, the width has an important impact on the resonant frequency of this vibration mode. The curve H = L=10 mm corresponds to the coupled vibration of the first-order flexural vibration around length and width directions. At this point, there is no longer an independent one-dimensional flexural vibration. In general, the conductance peak of this resonant frequency is higher than the conductance peak of an independent one-dimensional flexural vibration at the same size. Figure 11b shows that, when H/L is sufficiently small, the model more closely approaches the thin plate hypothesis, and the flexural vibration around one direction is not influenced by the other direction anymore. Moreover, the conductance peak in Figure 11a is much higher than the peak in Figure 11b. This is because flexural vibrations with smaller sizes are easier to return to their equilibrium position, causing lower mechanical internal resistance and a higher admittance value.

#### 3.1.3. Double-Laminated Vibrator with the Wedge Structure

**Figure 12:**
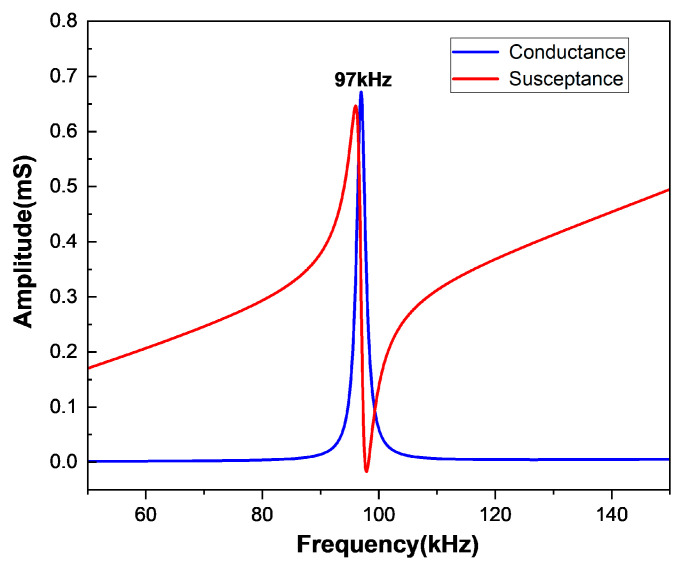
Admittance curve of rectangular double-laminated vibrator (L = 10 mm, W = 5 mm, H = 2 mm).

The working frequency of ultrasonic transducers in concrete detections is typically around 50–100 kHz. Based on the previous research of the two-dimensional and three-dimensional models, we chose the model parameters L = 10 mm, H = 4 mm, and W = 5 mm for further study. Figure 12 depicts the admittance curve within the frequency range of 50–150 kHz, and the resonant frequency of the first-order flexural vibration around the length direction is 97 kHz. Because the width is smaller than the length, the flexural vibration around the width direction mode is not generated inside this range, let alone the coupled vibration mode. The electromechanical coupling coefficient of this model is 0.231, which can be calculated by Equation (20).

**Figure 13:**
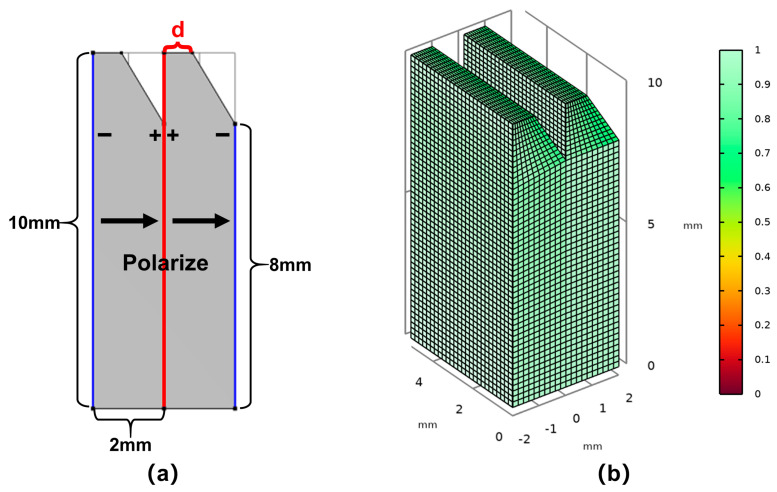
The double-laminated vibrator structure with the wedge angle: (**a**) dimension parameters, polarization, and electrodes of the model and (**b**) the model mesh quality.

**Figure 14:**
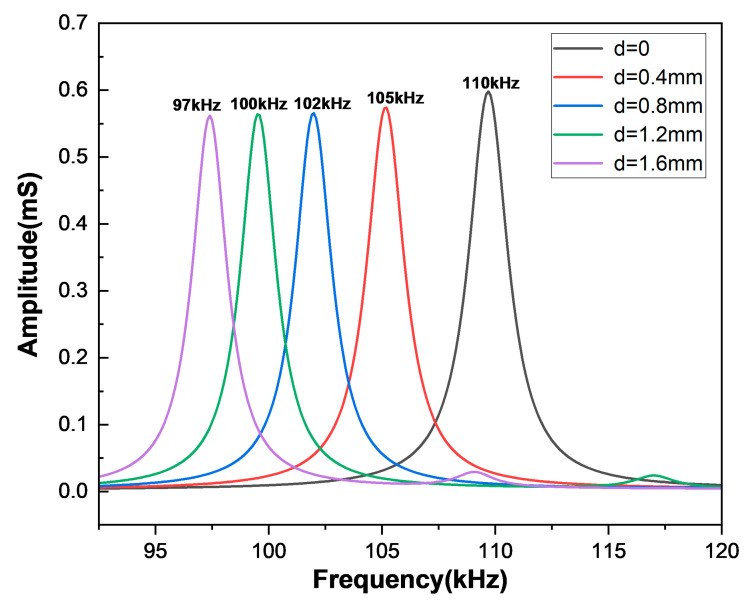
Conductance curves of the wedged double-laminated vibrator at different d parameters.

To prevent standing waves in the transducer’s backing layer, which are caused by the rectangular double-laminated structure, as well as to make it easier to lead electrode wires from the common surface in the preparation process, we changed the original rectangular structure to a double-laminated structure with wedge-shaped angles, as shown in Figure 13a. Figure 13b shows the mesh quality of this model. The upper edge of the wedge was set as parameter d, and the influence of d on the conductance curve of the double-laminated vibrator was studied. Figure 14 shows how the resonant frequency gradually increases within a small range as d reduces. When d = 0, the upper part of the model degenerates from a wedge shape to an edge shape, and at this point, the change in resonant frequency is the biggest. Considering the convenience of leading the electrode wires in the preparation process, a model size of d = 0.8 mm is an excellent choice for generating shear waves by flexural vibrations near 100 kHz, with an electromechanical coupling coefficient of 0.226 at this point.

### 3.2. Wavefield in Time Domain Based on Finite Element Analysis

Regarding the geometric dimensions of the wedge-shaped piezoelectric double-laminated vibrator discussed at the end of the previous section, we used the Comsol 6.2 finite element method to simulate the longitudinal and shear wave fields generated by the flexural vibration of the double-laminated vibrator in time-domain analysis. The acoustic wave propagation domain is a 400 m × 300 mm concrete layer with the density of 2300 $\mathrm{kg}/m^{3}$, a longitudinal wave speed of 3475 m/s, and a shear wave speed of 2128 m/s, and both sides of the concrete layer are absorption layers to reduce sound wave reflection at both interfaces. Since the purpose was only to briefly analyze the wave field, the matching layer and backing layer of the transducer were no longer included in this model. In the simulation, both sides of the wedge-shaped double-laminated vibrator were grounded, and a Gaussian-modulated sine pulse signal with a center frequency of 100 kHz was applied to the common surface, for which the form of the signal is $\sin\left(2\pi f_{0}t\right)\cdot e^{-{\left[2\left(t-2T_{0}\right)/T_{0}\right]}^{2}}$.

**Figure 15:**
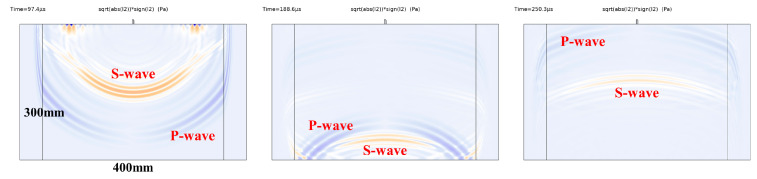
Wavefield of the double-laminated vibrator in the two-dimensional time domain model.

Figure 15 shows the acoustic wave field in the concrete layer of the two-dimensional model, with longitudinal waves and shear waves represented in the form of $\sqrt{\left|I_{2}\right|}\cdot sign\left(I_{2}\right)$, where $I_{2}=\frac{1}{2}\left(\sigma_{ii}\sigma_{jj}-\sigma_{ij}\sigma_{ij}\right)$ is the second invariant of stress, and σ is the stress. When the value of the formula is positive, it represents longitudinal waves, indicated by the blue part in the figure; when the value is negative, it represents shear waves, indicated by the yellow part in the figure. Figure 15 depicts the propagation directionality of longitudinal waves and shear waves, with shear waves concentrating in the central part and longitudinal waves propagating to both sides. There are almost no incident and reflected longitudinal waves propagating along the vertical central axis of the concrete layer. The reason for this wave propagation is that the electric field directions on the two individual piezoelectric plates of the vibrator are opposite, while the polarization directions are identical. As a result, vibrations of the longitudinal wave along the thickness direction, which are excited by two piezoelectric plates, have the opposite phase, leading to a tendency for mutual counteraction. Additionally, the longitudinal waves are weaker where they are closer to the central axis because of more counteraction. From the perspective of the probe structure, this effectively reduces the amplitude of the transducer’s received longitudinal wave signal, resulting in a favorable ratio of shear waves to longitudinal waves on the receiving terminal.

**Figure 16:**
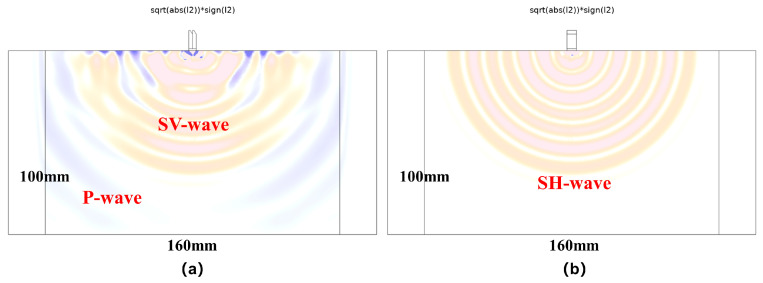
Wavefield cross-section of the three-dimensional time domain model of the double-laminated vibrator. (**a**) SV wave propagation cross section view; (**b**) SH wave propagation cross section view.

By extending the time-domain model to three dimensions, we can further observe the propagation of the shear horizontal wave field in another vertical section. Figure 16a depicts the wave field section inside the plane of the former two-dimensional model, while Figure 16b depicts wave field propagation in another vertical section. The shear horizontal waves generated by the flexural vibration of the double-laminated piezoelectric vibrator have strong omnidirectionality, enabling it to be more suitable for body wave detection on rock and concrete structures.

### 3.3. Discussion of Transducer Coupling Methods

**Figure 17:**
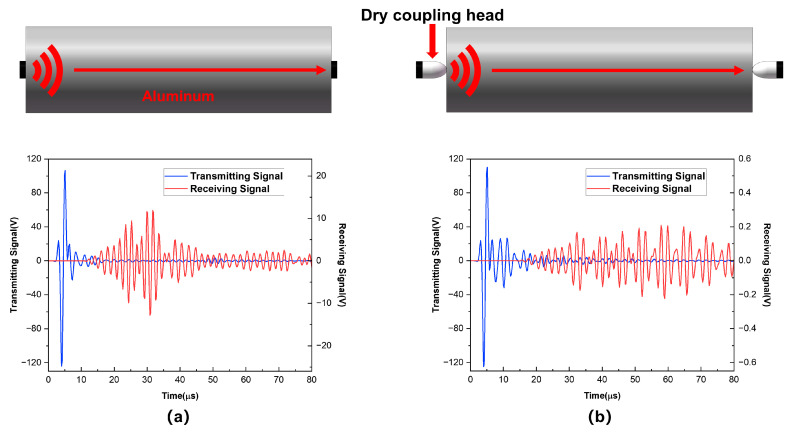
Models of different coupling methods and their signals: (**a**) surface coupling and (**b**) dry point coupling.

Consider a dry contact point coupling structure for the probe, which directly connects the piezoelectric vibrator to the dry-point-contact structure at the head of the probe. The dry point coupling method uses point contact instead of traditional surface contact, which generates substantially less acoustic energy than the traditional surface contact method. Therefore, in practical experiments, it is often used in an array configuration for parallel excitation. Figure 17a depicts a two-dimensional axisymmetric model, in which the top piezoelectric element emits a pulse signal that is received by the lower piezoelectric element through an aluminum cylinder with a height of 50 mm. In Figure 17b, an aluminum dry point coupling structure is connected to the transmitting and receiving piezoelectric elements. From two groups of received signals, the maximum amplitude of the received signal with surface coupling is 21 V, while adding a dry point coupling head greatly reduces the received signal to 0.34 V. Therefore, the acoustic energy of the dry point coupling transducer is far less than that of the surface coupling transducer.

**Figure 18:**
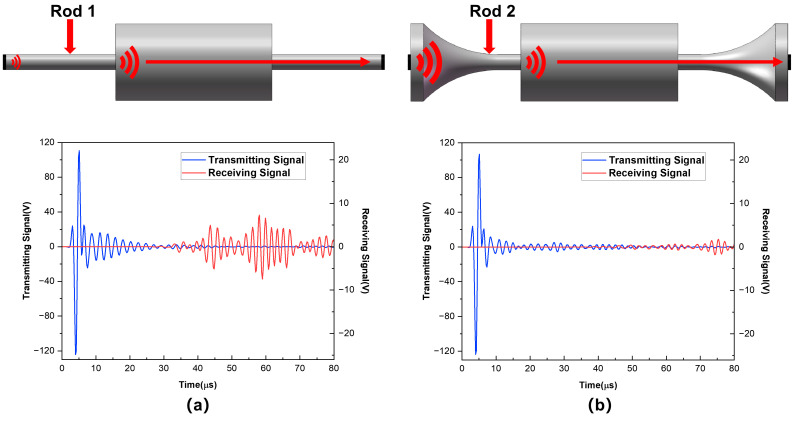
Two kinds of guide rod coupling models and their signals: (**a**) straight aluminum rod and (**b**) aluminum horn.

To significantly improve the acoustic energy of the dry-coupled transducer, we also simulated two kinds of aluminum rod configurations for dry coupling that both have a small front-end contact area, and their models and received signals are shown in Figure 18. Compared to the dry point coupling method, the maximum signal amplitudes of two-rod structure dry coupling methods are 8 V and 15 V, which are much bigger than those of the dry point coupling method. As a result, adopting a rod structure to accomplish dry coupling can increase the acoustic energy of the transducer.

## 4. Measurement Results

**Figure 19:**
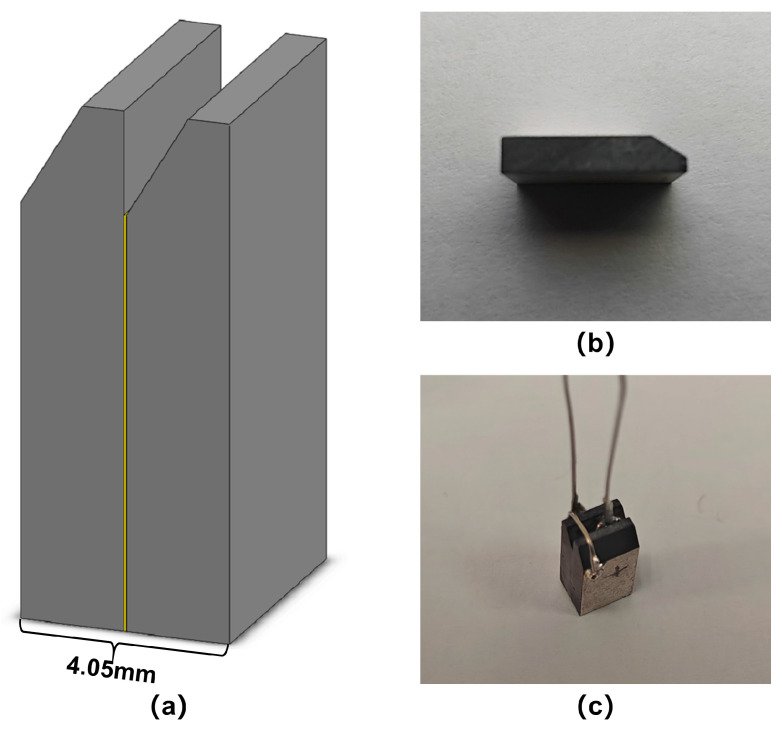
The piezoelectric material: (**a**) the double-laminated vibrator simulation model with an epoxy resin layer, (**b**) a single piezoelectric plate, and (**c**) the bonded piezoelectric double-laminated vibrator.

According to the optimization design of the size and shape of the piezoelectric double-laminated vibrator model from previous simulation results, we finally customized some piezoelectric plates, as shown in Figure 13, and used epoxy resin adhesive to bond two identical plates as the double-laminated vibrator. Then, we extended the negative and positive leads from side surfaces and the middle common surface. Figure 19 shows the single piezoelectric plate and the bonded double-laminated vibrator.

**Figure 20:**
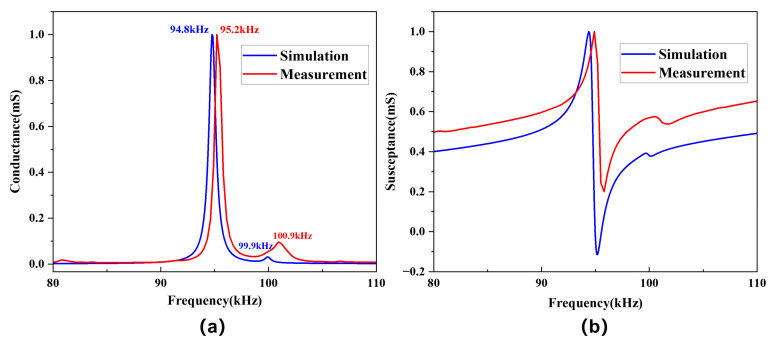
Comparison of the simulated and measured admittance of the double-laminated vibrator: (**a**) conductance and (**b**) susceptance.

In the bonding procedure, it was found that the thickness of the epoxy resin adhesive between two piezoelectric plates is approximately 0.05 mm. Therefore, we considered adding an epoxy resin layer with a 0.05 mm thickness to the wedge-shaped double-laminated vibrator simulation model to obtain a new admittance curve. On the other hand, we used an impedance analyzer (4294A, Agilent, Colorado Springs, CO, USA) to measure the actual admittance curve and then compared it with the simulation results, as shown in Figure 20. For the piezoelectric double-laminated vibrator model with the epoxy resin adhesive layer, Figure 20a demonstrates that the resonant frequency of the first-order flexural vibration is around 95 kHz, and the simulated results are in good agreement with measured ones. From the actual measured curve, we found another vibration mode near 100 kHz, which is also presented in the simulation results after adding the adhesive layer, further demonstrating that the simulation results are reliable. In our tests, we discovered that this vibration mode is related to the properties of the epoxy resin adhesive, but more studies are required to determine the specific relationship.

## 5. Discussions and Conclusions

Based on two-dimensional and three-dimensional rectangular piezoelectric double-laminated models, finite element simulations were conducted to optimize the size and shape of the vibrator. The general rule is that the resonant frequency of flexural vibration has a negative correlation with the length, while it has a positive correlation with the thickness of the vibrator. When the vibrator is sufficiently thin, its width has little impact on the resonant frequency because pure flexural vibrations occur only in the direction parallel to the flexural plane, and they are theoretically only affected by physical parameters parallel to the flexural plane. Thin plate models make it easier to excite pure flexural vibrations so that the width has almost no effect. However, if the length and width are similar in size, then their corresponding flexural vibrations around both directions are affected. In the practical application of this research, models with longer lengths than widths should be chosen as much as possible to avoid such coupling vibrations. From a theoretical perspective, all the fundamental principles of flexural vibrations around the length direction or the width direction are exactly alike. When the length and the width are entirely equal, the resonant frequency of the same order flexural vibrations around both directions are also completely the same, so there occurs only the coupled flexural vibration instead of in any single dimension. In addition, when vibrators are not thin enough, the theoretical values calculated by analytical formulas are no longer accurate, and the electromechanical coupling coefficient reduces with increasing vibrator’s whole thickness. The reason for this is that if the vibrator no longer approaches the thin plate model, then the assumptions in the theoretical derivation are not satisfied anymore, and the equation cannot be simplified. At this point, new methods need to be considered to solve the initial equations and obtain new expressions for the resonant frequency. Additionally, the increased thickness makes it more difficult for flexural vibrations to return to the equilibrium position, resulting in bigger internal mechanical resistance; thus, the conversion efficiency of mechanical energy to electrical energy in the piezoelectric vibrator also decreases. Through design optimization, we eventually determined the appropriate structure and dimensions of the double-laminated vibrator. Then, we customized the piezoelectric plates based on these parameters and bonded them into the double-laminated vibrator, measuring its admittance values. The measured results are in good agreement with the simulation ones, preliminarily proving the reliability of the simulation work.

On the other hand, the wave field propagation characteristics under the time-domain model of the double-laminated vibrator were simulated. Due to the characteristic of flexural vibrations where one piece expands and the other contracts, longitudinal waves tend to balance out in the central region, propagating to the sides, while shear waves are more concentrated in the middle region, and flexural vibrations can also generate omnidirectional SH waves. The high ratio of shear waves to longitudinal waves of the received signal and omnidirectional SH waves is beneficial for the subsequent imaging work of the transducer. Finally, a brief discussion on acoustic energy between the different coupling methods was conducted, discussing that the received signal using the dry point coupling structure is much weaker than using rod structure coupling because of the greatly reduced contact area. This has become the biggest challenge for optimizing the design of dry-coupled shear wave probes. In future studies, we may try to solve this problem by selecting piezoelectric materials that have larger electromechanical coupling coefficients, considering higher-order flexural vibration modes of double-laminated vibrators, which can generate higher acoustic energy, and using appropriate materials of the dry coupling head that have better acoustic impedance matching. Combined with the theoretical analysis and the simulation work of this paper, a good theoretical foundation has been laid for the subsequent preparation of the probe.

## Figures and Tables

**Table 1:** Parameters of the piezoelectric ceramics PZT-4.

Parameters	PZT-4	Parameters	PZT-4
$c_{11}^{E}(10^{10}\;N/m^{2})$	13.9	$\rho (\mathrm{kg}/m^{3})$	7500
$c_{12}^{E}(10^{10}\;N/m^{2})$	7.78	$d_{31}{(10}^{-12}\;C/N)$	−123
$c_{13}^{E}(10^{10}\;N/m^{2})$	7.43	$d_{33}{(10}^{-12}\;C/N)$	289
$c_{33}^{E}(10^{10}\;N/m^{2})$	11.5	$d_{15}{(10}^{-12}\;C/N)$	496
$c_{44}^{E}(10^{10}\;N/m^{2})$	2.56	$e_{31}(C/m^{2})$	−5.2
$s_{11}^{E}{(10}^{-12}\;m^{2}/N)$	12.3	$e_{33}(C/m^{2})$	15.1
$s_{12}^{E}{(10}^{-12}\;m^{2}/N)$	−4.05	$e_{15}(C/m^{2})$	12.7
$s_{13}^{E}{(10}^{-12}\;m^{2}/N)$	−5.31	$\varepsilon_{11}^{s}/\varepsilon_{0}$	730
$s_{33}^{E}{(10}^{-12}\;m^{2}/N)$	15.5	$\varepsilon_{33}^{s}/\varepsilon_{0}$	635
$s_{44}^{E}{(10}^{-12}\;m^{2}/N)$	39.0		

**Table 2:** Data comparison and relative error of listed analytical values and two-dimensional simulation values of the resonant frequency with a certain length.

L (mm)	H (mm)	Analytical Values (Hz)	Simulation Values (Hz)	Relative Error (%)
10	2	67,694	65,300	3.54%
	4	135,389	104,200	23.04%
	6	203,083	126,900	37.51%
	8	270,778	141,200	47.85%
	10	338,472	150,200	55.62%
20	2	16,924	17,900	5.45%
	4	33,847	32,700	3.39%
	6	50,771	43,800	13.73%
	8	67,694	52,100	23.04%
	10	84,618	58,500	30.87%
30	2	7522	8100	7.14%
	4	15,043	15,500	2.95%
	6	22,565	21,800	3.39%
	8	30,086	27,000	10.26%
	10	37,608	31,200	17.04%

**Table 3:** Data comparison and relative error of listed analytical values and two-dimensional simulation values of the resonant frequency with a certain thickness.

H (mm)	L (mm)	Analytical Values (Hz)	Simulation Values (Hz)	Relative Error (%)
2	6	188,040	156,100	16.99%
	12	47,010	47,000	0.02%
	18	20,893	21,900	4.60%
	24	11,753	12,600	6.72%
	30	7522	8100	7.14%
4	6	376,080	220,600	41.34%
	12	94,020	78,000	17.04%
	18	41,787	39,400	5.71%
	24	23,505	23,500	0.02%
	30	15,043	15,500	2.95%

## Data Availability

Data is contained within the article.
